# Landscape complexity and agricultural pressure at the habitat-level scale determine impact of insecticide use on future generation of non-target arthropods (ground beetle *Poecilus cupreus*)

**DOI:** 10.1007/s10646-025-02995-5

**Published:** 2026-01-03

**Authors:** Grzegorz Sowa, Elżbieta Ziółkowska, Zuzanna M. Filipiak

**Affiliations:** Institute of Environmental Sciences, Faculty of Biology, Jagiellonian University, Gronostajowa 7, 30-387, Krakow, Poland

**Keywords:** Carabidae, Pulse exposure, Insecticides, Landscape ecology, Susceptibility, Biomarker

## Abstract

Agricultural intensification, particularly pesticide use, has reduced beneficial organisms and their functional diversity in agroecosystems. We studied the effects of pulsed exposure to two common insecticides, Mospilan 20 SP (neonicotinoid - active ingredient (a.i.) acetamiprid) and Sherpa 100 EC (pyrethroid - a.i. cypermethrin), on the non-target ground beetle *Poecilus cupreus* (Coleoptera: Carabidae) in a large-scale landscape context. Laboratory bred beetles (F2) from two agricultural landscapes of varying complexity (low vs. high) and two habitat types with differing pesticide pressure (crops vs. meadows) were tested. We hypothesized that beetles from high agricultural pressure habitats would exhibit reduced susceptibility to insecticides due to adaptations from repeated exposures, with stronger effects in low-complexity landscapes. Insecticide response depended on landscape complexity and habitat type. Beetles from high-pressure habitats showed reduced sensitivity in low-complexity landscapes. Mortality was higher in low-pressure habitats within low-complexity landscapes. Sherpa exposure caused sublethal effects on acetylcholinesterase (AChE) activity, influenced by prior insecticide exposure. Control beetles from high-pressure habitats exhibited higher initial AChE activity than those from low-pressure habitats. Sherpa exposure increased AChE activity in beetles from low-pressure habitats but decreased it in those from high-pressure habitats. Our findings emphasize that insecticide effects are context-dependent, influenced by landscape complexity, habitat type, and prior exposure. They underscore the importance of considering long-term, multi-generational perspectives in ecotoxicological studies and augmentative pest management.

**Supplementary Information:**

The online version contains supplementary material available at 10.1007/s10646-025-02995-5.

## Introduction

The excessive use of pesticides, the conversion of non-crop areas to agricultural use, and the homogenization of landscapes are aspects of agricultural intensification. These factors have led to the loss of species and a reduction in their functional diversity worldwide in recent decades (e.g., Raven and Wagner 2021; Tilman et al. 2001). Among these, pesticides are one of the greatest threats to beneficial insects in agricultural landscapes, not only because of the direct mortality they cause, but also because of various sub-lethal effects, including effects on insect physiology and behaviour (Desneux et al. 2007).

Crops can be treated subsequently with pesticides several times during the growing season (Frische et al. 2018). In fact, it is a common practice to apply different insecticides in a timed sequence, e.g. pulsed spraying, where insecticides are applied to the crop more than once during the recommended period, with a specified interval between each application (Kanu et al. 2021). Repeated applications of insecticides are expected to be more harmful to organisms than a single application, as each subsequent application would eliminate part of the population (Andersen et al. 2006; Liess et al. 2013). As insecticides are reapplied, their residues accumulate in the environment, leading to increased exposure for both target species and those that are not the intended targets of a pesticide, pest control method, or other human action but are unintentionally affected by it (non-target organisms). The toxic compounds present in these insecticides can persist in ecosystems, affecting a range of organisms. Consequently, the ecological balance becomes disrupted as various species experience sustained pressure from the chemical compounds introduced through repeated applications. Moreover, the toxic effects of pulse exposures can be magnified if organisms do not fully recover between pulses, with mortality rates increasing with each pulse (Kanu et al. 2021; Wiberg-Larsen et al. 2021).

A mechanistic understanding is key for linking insecticide exposure to effects at both individual and population levels. In arthropods, resistance and tolerance often involve altered detoxification pathways or reduced cuticular penetration, modifying internal dose–response relationships. These physiological traits interact with ecological context – such as spatial heterogeneity in exposure, potentially accelerating the evolution of resistance, even in non-target species. While most existing research has focused on pest species (Hawkins et al. 2019), non-target arthropods may respond differently to insecticides due to traits such as mobility or feeding guild (Bras et al. 2022; Torres et al. 2015). A recent global meta-analysis confirmed that pesticides negatively affect a broad spectrum of non-target organisms, regardless of taxonomic group or ecosystem type, highlighting the potential for widespread sublethal and transgenerational effects (Wan et al. 2025). Moreover, the consequences of past pesticide exposure may extend beyond immediate mortality, manifesting as altered physiological and biological processes that affect life-history traits or concentration of metabolites in subsequent generations (Clements et al. 2020; Poulsen et al. 2021). Laboratory studies continue to provide valuable mechanistic insights into insecticide effects on both pest and beneficial insects, but they typically rely on single-generation or laboratory-based designs. For example, a recent study on *Eriopis connexa* demonstrated that insecticide mixtures can override physiological resistance, while resistant populations of *Spodoptera litura* may exhibit elevated detoxification enzyme activity and oxidative stress (Mahajan and Kaur 2025; Soares et al. 2025). However, such approaches often overlook the influence of historical exposure and landscape structure of the species’ habitat origin, as well as multi-generational adaptation in shaping species’ responses to pesticides.

Insecticide responses in arthropod populations within agroecosystems are shaped by both prior pesticide exposure and landscape complexity, which encompasses habitat composition (i.e., the proportion of land cover or land use types), configuration (i.e., spatial arrangement such as fragmentation or interspersion), and heterogeneity (i.e., habitat richness and evenness) (Estrada-Carmona et al. 2022; Martin et al. 2019). Repeated exposure can lead to resistance or cross-resistance in both pests and non-target species (Torres et al. 2015; Willis et al. 2020), with resistance more likely to emerge in homogeneous landscapes where limited gene flow constrains genetic variability (Riggi et al. 2016). In contrast, complex landscapes – characterized by diverse habitat composition, configuration, and heterogeneity – can facilitate gene flow and potentially mitigate resistance development (Lozier et al. 2013; Yadav et al. 2019). Pesticide use, together with annual changes in crop mosaics, results in spatially and temporally heterogeneous exposure patterns for species inhabiting agroecosystems. The European Food Safety Authority (EFSA 2015) has emphasized the importance of incorporating landscape context into pesticide risk assessments of non-target arthropods, and recent studies confirm that landscape complexity influences insect abundance and diversity (Martin et al. 2019; Triquet et al. 2023). However, the role of landscape complexity in shaping pesticide tolerance in non-target species, especially under chronic exposure, remains poorly understood (Bras et al. 2022; Sowa et al. 2022).

To develop more effective agricultural management and protection strategies for non-target arthropods, it is essential to better understand: (1) the toxic effects of commonly used insecticides, typically applied in the form of short-term pulses, and (2) how these effects vary among arthropod populations exposed to different conditions at the local habitat and landscape scales. Assessing pesticide sensitivity in populations of beneficial non-target arthropods found in agricultural landscapes of varying complexity is crucial for informing future mitigation strategies and ecotoxicological testing (Levine et al. 2019; Boyd et al. 2025). Our study addresses this gap by applying an ecotoxicological framework that integrates landscape-scale ecological variables with organism-level toxicological endpoints to evaluate how environmental context influences susceptibility to insecticides.

We focused on the ground beetle *Poecilus cupreus* (Coleoptera: Carabidae) (Linnaeus, 1758), a widespread and important predatory non-target beneficial arthropod in European agricultural ecosystems, contributing to natural pest control (Desender et al. 1994; EFSA 2015). Using pulse-exposure scenarios, we evaluated both lethal (survival probability) and sublethal responses, i.e., acetylcholinesterase (AChE) activity. To account for the multi-generational perspective, the second generation (hereafter F2) of beetles was tested, which parental generation (hereafter P) originated from habitats with different levels of agricultural pressure (low versus high pesticide exposure) and from two distinct landscapes with different levels of complexity (low versus high complexity). We predicted that different schemes of insecticide applications (i.e., single vs. pulse exposure), and levels of agricultural pressure and landscape complexity would affect beetles differently. We hypothesized: (i) lower probability of survival after second pulse than after first pulse of exposure due to slow recuperation; (ii) higher probability of survival for beetles originated from habitats with high agricultural pressure, due to long history of insecticide use (preselection for resistance/tolerance) regardless of the landscape complexity; (iii) resistance, if present, would be more evident in individuals originating from landscape with lower complexity, because resistance can prevail in large spatially homogeneous areas; (iv) AChE activity is expected to be higher in beetles from high compared to low agricultural pressure habitats, especially under insecticide treatments, and differences should be more pronounced in low complexity landscape. To the best of our knowledge, this is the first study to assess the effects of pulsed insecticide exposure on a non-target arthropod by explicitly linking physiological responses to environmental variables such as habitat origin and landscape complexity, thereby enhancing the ecological realism of ecotoxicological risk assessment.

## Materials and methods

### Research area

**Fig. 1:**
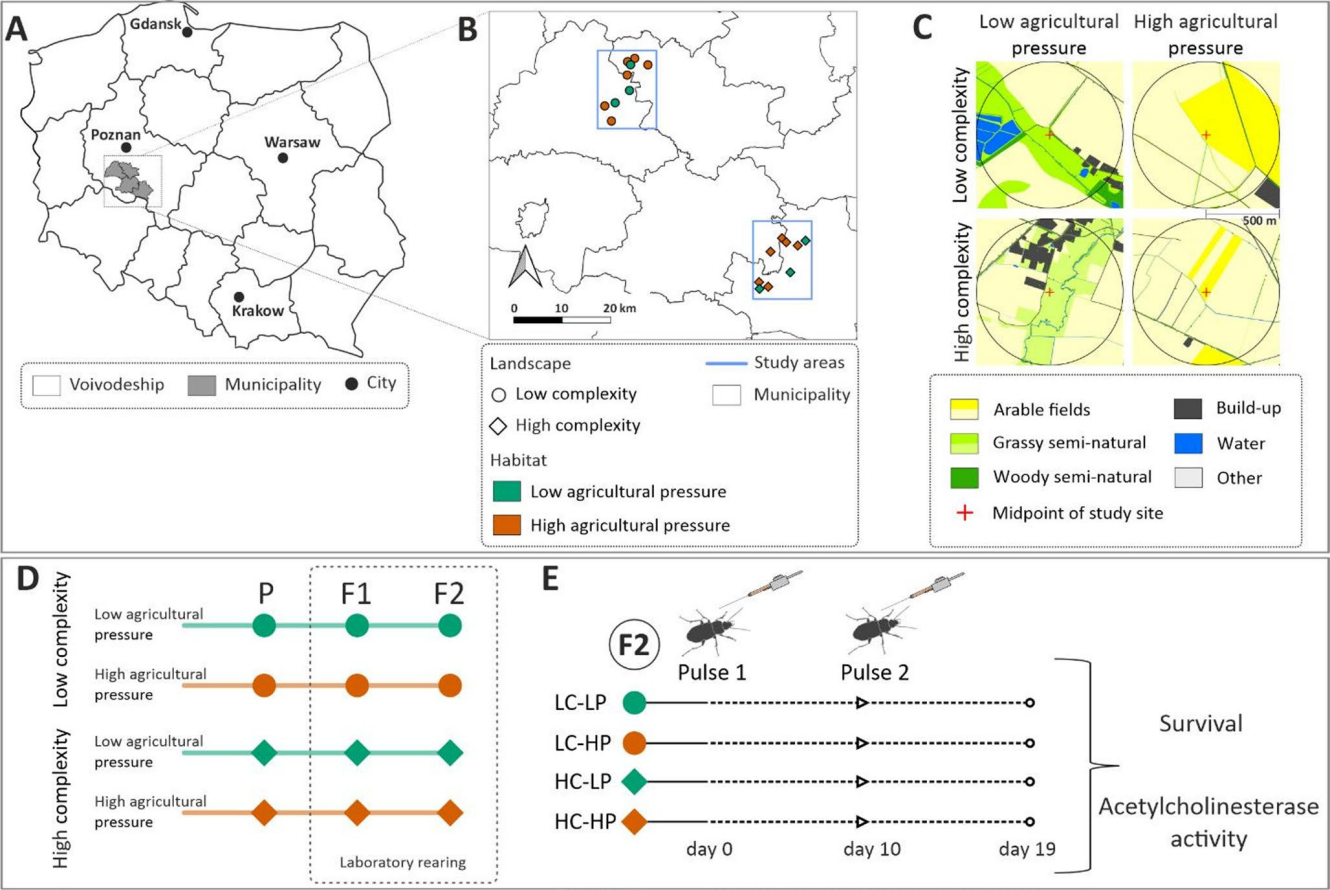
Location of the study areas within the Wielkopolska Voivodeship, Poland (**A**). Six sites classified as habitats of high agricultural pressure (HP; orange symbol) and three sites classified as habitats of low agricultural pressure (LP; green symbol) were selected within two distinct study areas (**B**) – the low complexity landscape (LC; circle symbol) and the high complexity landscape (HC; diamond symbol). Land cover for exemplary sites representing both types of landscapes and habitats (**C**). Darker yellow colour in the C panel represents oilseed rape coverage. Within the midpoint of each study site (red cross symbol), traps were set up (8 × 8 m, 64 m^2^) for collection of the parental generation of ground beetles *Poecilus cupreus*. Parental beetles (P) were collected and reared under laboratory conditions for two generations (F1 and F2) (**D**). Individuals from the F2 generation were exposed to two sequential pulses of the insecticides Mospilan 20 SP (a.i. acetamiprid) and Sherpa 100 EC (a.i. cypermethrin). Survival was assessed after each pulse, and acetylcholinesterase activity was measured after the second exposure

The P generation was collected from a typical agricultural area located in western Poland (Fig. 1). Within this region, two distinct landscapes (each 12 × 16 km; approx. 192 km^2^) of varying complexity were selected based on analysis of high-resolution (1 m^2^) land cover maps (see Sowa et al. 2022 for details). The low-complexity landscape (hereafter LC) comprises predominantly medium to large fields (fields > 10 ha covering over 50% of the area), primarily managed by large-scale commercial agricultural enterprises. The high-complexity landscape (hereafter HC), in contrast, features small, fragmented fields (< 3 ha covering > 40% of the area). This landscape exhibits greater heterogeneity in land use and a higher density of field margins, consistent with traditional family farming systems (Table S1). Importantly, both landscapes share comparable pedo-climatic conditions and have similar overall proportion of arable land, so that the density of field boundaries is much higher in the HC landscape than in the LC landscape.

Within each landscape, nine study sites per landscape were selected and further classified into high or low agricultural pressure habitats, based on land use composition within a 500 m radius from the trap midpoint (corresponding to an area of ca. 0.785 km^2^). This spatial scale is biologically relevant for *P. cupreus*, as it approximates the species’ typical lifetime dispersal range. The habitats defined in this manner were predominantly composed of land cover types preferred by *P. cupreus* (open areas of fields, meadows and pastures) (Fig. 1C). However, they differed in terms of agricultural pressure estimated based on proportion of arable land (including crop type) relative to non-arable land. Higher proportions of intensively managed crops are indicative of increased pesticide pressure. Sites with high agricultural pressure (hereafter HP) were located within winter oilseed rape fields – a crop known for its high pesticide input, typically receiving between one and five insecticide treatments per growing season (Richardson 2008), particularly during spring when *P. cupreus* is most active. In HP sites, arable land comprised between 64% and 98% of the surrounding habitat (on average: 88%), with oilseed rape dominating the arable area, accounting for 10% to 100% at the time of beetle sampling. Non-arable open habitats, such as permanent grasslands or meadows, were in minority (0–25%, on average: 5%; Table S2). Conversely, low-pressure sites (hereafter LP) were located in permanent grasslands managed with minimal disturbance (e.g., mowing without pesticide application). These sites exhibited a higher proportion of non-arable open habitats (10–31%, on average: 21%), while arable land constituted 46–73% of the area (on average: 60%; Table S2). Importantly, LP sites lacked oilseed rape, and other intensively treated crops were rare (e.g., winter wheat covered < 7% of arable land), resulting in substantially reduced direct pesticide exposure.

In each landscape, six study sites were distinguished that were classified as habitats of high agricultural pressure and three that were classified as habitats of low agricultural pressure. Due to logistical constraints and the fact that beetle populations were much higher in habitats of low than of high agricultural pressure, only three sites in the former were selected for sampling.

### Test organisms

Sixty-four traps were placed at the midpoint of each study site in a grid of 64 m^2^ (8 × 8 m). Study sites were spaced at least 800 m apart to avoid sampling the same beetle population, but no more than 60 km apart to maintain comparable climate and edaphic conditions. Adult *P. cupreus* (P generation) were collected with pitfall traps in April and May 2018. Beetles collected in each habitat type of each landscape were cultured in the laboratory in four groups representing the types of landscapes/habitats from which they originated, i.e., LC-HP (low complexity – high agricultural pressure), LC-LP (low complexity – low agricultural pressure), HC-HP (high complexity – high agricultural pressure), HC-LP (high complexity – low agricultural pressure). The collected beetles were kept in a climatic chamber at 20 °C and 70% relative humidity (RH) under a 16:8 light: dark (L: D) h regime, in plastic boxes (ca. 1000 mL) filled with moist peat and fed *ad libitum* with artificial food made of ground mealworms mixed with minced apple in a ratio of 7:3 (w: w). The beetles were cultured for the next two generations (F1 and F2), i.e., for two consecutive years, according to Sowa et al. (2022) (Fig. 1D).

### Laboratory experiment design and insecticides used

The F2 beetles tested (*n* = 309) were a mixture of adult males and females. The number of beetles tested per landscape/habitat group (as defined above) varied depending on beetle availability. For each insecticide treatment and control, we used the following numbers of beetles: *n* = 35 × 3 = 105 in total for LC-LP, *n* = 21 × 3 = 63 in total for LC-HP, *n* = 17 × 3 = 51 in total for HC-LP, and *n* = 30 × 3 = 90 in total for HC-HP group. Before the start of the experiment, beetles were weighed to the nearest 0.0001 g and placed in empty plastic cups (diameter: 70 mm, height: 44 mm) with perforated lids and moisturize filter papers on the bottom for a 24 h acclimatization period. The test concentrations of the pesticides were chosen based on the preliminary study in which the LD_50_ (i.e. causing 50% mortality; 48 h) with corresponding 95% confidence intervals were estimated using probit analysis. Full details of the preliminary experiment can be found in the *Supplementary Information* and Table S3.

We exposed the F2 beetles to commercially available insecticide formulations commonly used in the study area (according to a questionnaire we conducted among local farmers), namely, Mospilan 20 SP (Nippon Soda, Japan; referred to as Mospilan) with active ingredient (a.i.) acetamiprid and Sherpa 100 EC (SBM Developpement SAS, France; referred to as Sherpa) with a.i. cypermethrin (for more information on selected insecticides see *Supplementary Information*). The selected insecticides have active ingredients with different modes of action and therefore belong to different insecticide groups; Mospilan 20 SP is a neonicotinoid, while Sherpa 100 EC is a pyrethroid. Insecticides in their commercial form were dissolved in acetone as a carrier to obtain LD_50_ (48 h) concentrations corresponding to: 9.2 recommended concentration for field use (RCF; 4.3–23.2; 95% confidence interval) for Mospilan and 1.2 RCF (0.9–1.6; 95% confidence interval) for Sherpa. The chosen RCF corresponded to 0.74 µg a.i. × µL^− 1^ for Mospilan and 0.12 µg a.i. × µL^− 1^ for Sherpa. The LD_50_ value for Mospilan could not be accurately determined, possibly due to low toxicity to the selected species or other unknown reasons. In other studies, LC_50_ or LD_50_ estimates for insecticide formulations containing acetamiprid also showed relatively large confidence intervals (Biddinger et al. 2013; Mokkapati et al. 2021). For example, after topical exposure to Mospilan, the estimated LC_50_ (48 h) for the solitary bee *Osmia bicornis* was 39.9 in RCF with a wide 95% confidence interval of 28.9–73.2 RCF (Mokkapati et al. 2021). To account for this inaccuracy in our study, in the following parts of the manuscript we will refer to concentrations near-LD_50_ rather than the LD_50_ itself for Mospilan. Consequently, the results obtained for the two insecticides were not compared with each other, meaning the analyses for both insecticides were performed separately.

The beetles from each group, i.e., LC-LP, LC-HP, HC-LP, HC-HP, were assigned to three treatments (Mospilan, Sherpa, and control). The experiment lasted for 19 days, and the respective insecticides were applied on day 0 (first exposure pulse) and day 10 (second exposure pulse) (Fig. 1E). Beetles found dead after the first pulse were not included in the second pulse. Beetles were exposed individually to 1 µl of insecticide solution, while individuals from the control groups were treated with acetone alone. Solutions were applied on the scutellum using a repeating topical dispenser attached to Hamilton syringe (Hamilton company, USA). Beetles in plastic cups were kept at 20 °C and 75% RH under a 16:8 (L: D) h cycle.

### Observations and measurements

We used the following traits to investigate the susceptibility of F2 beetles to insecticides: body mass, survival, and AChE activity. Survival was studied because the concentrations used were estimated to cause 50% mortality. Body mass may influence susceptibility to insecticides because the smaller the individual, the more insecticide it will take in per unit of body weight. Acetylcholinesterase enzymatic activity was investigated as a potential biomarker of previous generations exposure to insecticides. Inhibition of AChE activity is expected following exposure to organophosphates and carbamates (Fulton and Key 2001). However, some studies have shown that AChE activity in invertebrates can also be altered (both increase and decrease was observed) following exposure to other groups of insecticides (Badiou and Belzunces 2008), including pyrethroids (Misiewicz et al. 2024;) and neonicotinoids (Li et al. 2017).

#### Recording the mortality

Mortality was recorded at 3, 5, 24 h and daily afterwards after each exposure to insecticide or acetone (i.e., on days 0 and 10) till the end of experiment at day 19. Beetles were classified as dead after being gently prodding with a pair of tweezers, followed by observation of their movements. To increase the ecological validity of the study, where immobilized beetles are vulnerable to environmental factors and predation, any beetle observed to be knocked-down (unable to walk or relocate) during the survival analysis was considered to be ecologically dead. Mortality was monitored in the control treatment to exclude that mortality was caused by factors other than the effect of the insecticides. At the end of the experiment (at day 19), surviving beetles were weighed again, frozen in liquid nitrogen, and stored at − 80 °C until further analysis.

#### Preparation of tissue extracts

Whole beetles were prepared for homogenization on ice in micro tubes containing 1 mL 0.05 M phosphate buffer (pH 7.4) comprising Trition X-100 at a 1:4 tissue: buffer (w: v) ratio and five pieces of 2.8 mm ceramic bead media (Omni International, USA). Samples were homogenised for 1 min using a mechanical homogeniser (Bead Ruptor Elite, Omni International, USA). Homogenates were centrifuged at 4 °C for 15 min at 15,000 *g* (MPW-350R, MPW MED Instruments, Poland) and the supernatant from each sample was transferred to a new 2 mL tube and used for AChE activity and protein analyses.

#### Acetylcholinesterase and total protein assay

Due to the high mortality of individuals exposed to Mospilan insecticide, resulting in a small sample size, analyses on AChE activity were only performed on beetles exposed to Sherpa insecticide using the following numbers of beetles: LC-LP – 32 (control treatment); 15 (Sherpa treatment), LC-HP – 20 (control treatment); 15 (Sherpa treatment), HC-LP 14 (control treatment); 11 (Sherpa treatment) and HC-HP 29 (control treatment); 16 (Sherpa treatment). Acetylcholinesterase activity was measured according to the generic method of Ellman et al. (1961) on 96-well plates (Sarstedt, USA), using the Gen5™ spectrometer (BioTek^®^ Instruments, USA). A reaction medium was prepared for each sample by mixing 10 µL of supernatant, along with 175 µL phosphate buffer (0.05 M, pH 7.4), and 10 µL of 0.01 M DTNB solution prepared in the 0.1 M Tris-HCl. The reaction was initiated by adding 5 µL solution of 0.1 M acetylthiocholine iodide mixed with double distilled water as a substrate. The absorbance was measured after 1:30 min incubation for 4:20 min at 405 nm. The reaction medium containing phosphate buffer (0.05 M, pH 7.4) was used as a negative control. AChE activity was expressed as nanomoles of acetylcholine hydrolysed per minute per mg protein (nmoles min^− 1^ mg^− 1^ protein).

The protein content of the same supernatant as used for AChE analysis was assessed using the same appliances. The homogenate was mixed with Bradford’s reagent (1:50 homogenate: reagent ratio), incubated for 15 min at 25 ℃ and then the absorbance was measured at 600 nm using BSA as a standard. Protein content was expressed as mg g^− 1^ beetle (fresh weight). Protein analysis was necessary for the final assessment of AChE activity.

### Data analyses

In our study design, beetle populations from contrasting environments were reared under standardised laboratory conditions for multiple generations to isolate inherited differences associated with their origin (cf. common garden approach in Brans et al. (2021)). Thus, the landscape type × habitat type combination was used as a categorical proxy for lineage-specific environmental history, and each group was treated as an experimental unit with shared ancestry reflecting the environmental history of its source population.

To assess the pulse-dependent and origin-specific effect of insecticide exposure on beetle survival, logistic regression models were fitted separately for each insecticide. Due to the small sample size and the separation in the data, we applied a Firth’s bias-reduced penalized likelihood logistic regression which overcomes these issues (‘logistf’ package; Heinze and Schemper 2002). The following effects were tested: habitat type, landscape type, pulse, pulse × habitat type, pulse × landscape type, landscape type × habitat type. To control for possible differences in susceptibility to the insecticides, body mass was included as a covariate. The analysis started with the formulation of the full model, i.e., all main factors and interactions were tested for significance (*p* ≤ 0.05). If body mass was non-significant, it was removed from the model, and then a backwards selection with retained lower order effect was used to remove non-significant factors (and/or their interaction(s) from the model. Model significance was assessed using a penalized likelihood ratio test. For interactive effects, post-hoc analyses were then performed using ‘emmeans-logistf’ function (Heinze et al. 2023) from the ‘emmeans’ package (Lenth 2018).

To determine the effect of exposure to Sherpa on AChE activity, a generalized linear model (GLM) with Gaussian distribution was fitted, testing the following effects: habitat type, landscape type, insecticide treatment, habitat type × insecticide treatment, landscape type × insecticide treatment, landscape type × habitat type. To assess effect significance, the single model with the lowest Akaike Information Criterion corrected for small sample sizes (AICc) was selected using ‘MuMIn’ package (Bartoń 2022;), followed by the function ANOVA from the ‘car’ package to obtain *p*-values (Fox and Weisberg 2019). Post hoc analyses were then performed by calculating estimated marginal means by using the ‘emmeans’ package (Lenth 2018). All analyses were performed using R software, version 4.2.1. (R Core Team 2022).

## Results

### Beetles response to estimated LD_50_

Variability in beetle mortality was observed among the groups after the first insecticide pulse. The observed mortality rates did not always correspond to the expected mortality rate of 50%. In the case of Sherpa, the first pulse of the applied LD_50_ concentration resulted in the expected mortality only in the beetles from habitats of high agricultural pressure from high complexity landscape (HC-HP) (47% mortality), whereas 37%, 29% and 10% mortality were observed for beetles from habitats of low agricultural pressure from low complexity landscape (LC-LP), of low agricultural pressure from high complexity landscape (HC-LP), and of high agricultural pressure from low complexity landscape (LC-HP), respectively. For Mospilan, the first pulse applied at a near-LD_50_ concentration resulted in the expected mortality in three groups, i.e. 47% in HC-HP, 52% in LC-HP, 53% in HC-LP, while the mortality in the LC-LP group was 94%.

### Survival after exposure to Mospilan

**Fig. 2:**
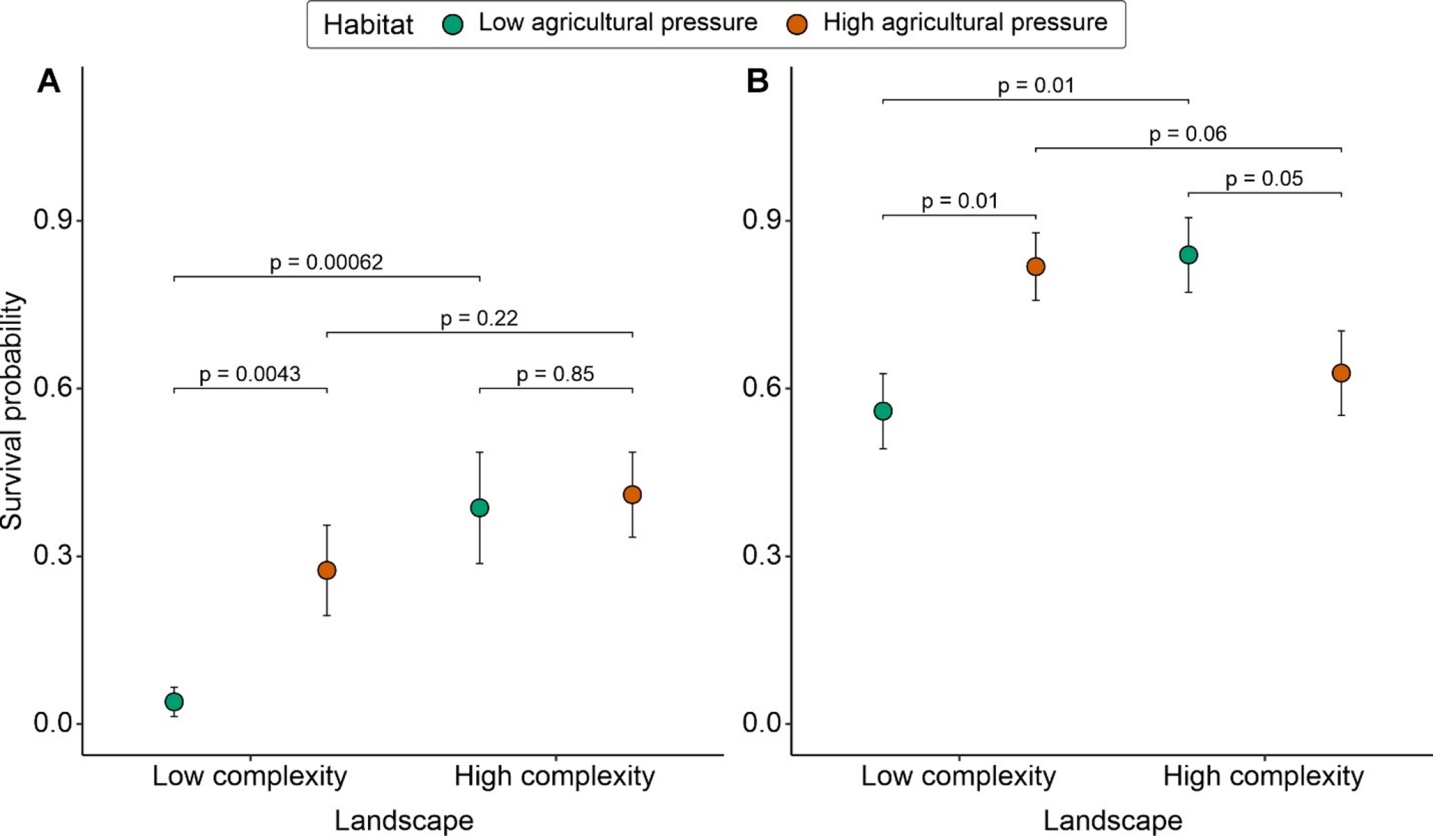
The effect of habitat and landscape type on probability survival of *Poecilus cupreus* beetles (F2 generation) at the end of the experiment (day 19) after pulsed exposure to 48 h near-LD_50_ concentration of Mospilan (**A**) (9.2 RCF; 0.74 µg a.i. × µL^− 1^; a.i. acetamiprid) or 48 h LD_50_ concentration of Sherpa (**B**) (1.2 RCF; 0.12 µg a.i. × µL^− 1^; a.i. cypermethrin) insecticides. RCF – Recommended Concentration for Field use. Points represent estimated probabilities and bars represent standard errors

The final Firth logistic regression model, i.e., after backward selection with retained lower order effect (i.e., habitat type, landscape type, pulse), for beetle survival after exposure to Mospilan is presented in Table 1. The survival of beetles after exposure to Mospilan was influenced by: (1) habitat type (coefficient = -2.219, SE = 0.765, *p* = 0.001), (2) pulse (coefficient = -1.152, SE = 0.459, *p* = 0.009), and (3) interaction of habitat type and landscape type (coefficient = 2.120, SE = 0.915, *p* = 0.01) (Table 1). The survival rate of beetles after the second pulse was 22%, significantly lower than the survival rate after the first pulse, which reached 35%. A post hoc analysis on the interaction between habitat type and landscape type showed that, within the low complexity landscape (LC) the survival of *P. cupreus* was 27% higher for beetles from habitats of high agricultural pressure (HP) compared to those from habitats of low agricultural pressure (LP) (t-ratio = -2.901, df = 134, *p* = 0.0043; Fig. 2A), whereas within high complexity landscape (HC) survival was similar for beetles from both habitat types (t-ratio = -0.194, df = 134, *p* = 0.85; Fig. 2A). Beetles from high complexity landscape (HC) were 39% more likely to survive than beetles from low complexity landscape (LC), but only if they originated from habitats of low agricultural pressure (LP) (t-ratio = 3.504, df = 134, *p* = 0.00062; Fig. 2A), and not from habitats of high agricultural pressure (HP) (t-ratio = 1.239, df = 134, *p* = 0.22; Fig. 2A).

**Table 1:** Results of the final firth’s bias-reduced logistic regression models for overall survival of *Poecilus cupreus* beetles (F2) originating from different landscape and habitat types after pulse exposure to Mospilan or Sherpa insecticides. The model coefficients along with standard errors (SE), the 95% confidence intervals (95% CI), chi-square statistics (χ2), and p-values are presented

Insecticide	Model term	Coefficient	SE	95% CI	χ2	*p*-value
Mospilan^1^	Intercept	0.758	0.681	(-0.579, 2.113)	1.239	0.3
	Habitat type	-2.219	0.765	(-3.953, -0.841)	10.644	0.001
	Landscape type	0.608	0.490	(-0.342, 1.598)	1.559	0.2
	Pulse	-1.152	0.459	(-2.104, -0.280)	6.818	0.009
	Habitat type × Landscape type	2.120	0.915	(0.410, 4.086)	6.000	0.01
Sherpa^2^	Intercept	2.552	0.826	(1.001, 4.280)	10.947	0.0009
	Habitat type	-1.264	0.492	(-2.290, -0.337)	7.302	0.007
	Landscape type	-3.609	1.145	(-5.940, -1.410)	10.602	0.001
	Pulse	-0.699	0.454	(-1.611, 0.187)	2.387	0.1
	Habitat type × Landscape type	2.394	0.747	(0.968, 3.933)	11.197	0.0008
	Landscape type × Pulse	1.751	0.725	(0.355, 3.234)	6.097	0.01

^1^Likelihood ratio test = 27.045 on 4 df, *p* = 0.00002, *n* = 139; ^2^Likelihood ratio test = 17.247 on 5 df, *p* = 0.004, *n* = 172.

### Survival after exposure to Sherpa

The final Firth logistic regression model, i.e., after backward selection with retained lower order effect (i.e., habitat type, landscape type, pulse), for beetle survival after exposure to Sherpa is presented in Table 1. The survival of beetles after exposure to Sherpa was influenced by: (1) habitat type (coefficient = -1.264, SE = 0.492, *p* = 0.007), (2) landscape type (coefficient = -3.609, SE = 1.145, *p* = 0.001), (3) interaction of habitat type and landscape type (coefficient = 2.394, SE = 0.747, *p* = 0.0008), and (4) moderately by the interaction of landscape type and pulse (coefficient = 1.751, SE = 0.725, *p* = 0.01) (Table 1). A post hoc analysis of the interaction between habitat type and landscape type showed that, within the low complexity landscape (LC), beetles from habitats of high agricultural pressure (HP) survived moderately better than beetles from habitats of low agricultural pressure (LP) (t-ratio = -2.572, df = 166, *p* = 0.01; Fig. 2B). Within the high complexity landscape (HC), they survived marginally better when originating from habitats of low (LP) than high (HP) agricultural pressure (t-ratio = 2.006, df = 166, *p* = 0.05; Fig. 2B). Survival was 25% higher for beetles from the habitats of low agricultural pressure (LP) in high complexity landscape (HC) compared to beetles from the same habitat type in low complexity landscape (LC) (t-ratio = 2.504, df = 166, *p* = 0.01; Fig. 2B), whereas marginally higher survival was observed for beetles originated from the habitats of high agricultural pressure (HP) in low complexity (LC) compared to high complexity (HC) landscape (t-ratio = -1.892, df = 166, *p* = 0.06; Fig. 2B). A post hoc analysis on the interaction between landscape type and pulse showed that the survival of beetles from the high complexity landscape (HC) was 21% higher than that of beetles from the low complexity landscape (LC) after the second pulse of Sherpa, but the difference did not reach statistical significance (t-ratio = 1.823, df = 166, *p* = 0.07). In turn, no differences were observed after the first pulse of Sherpa between landscape types (t-ratio = -1.431, df = 166, *p* = 0.15). Comparison between pulses revealed that survival of beetles from high complexity landscape (HC) was 23% higher after the second pulse compared to the first, but the observed difference was marginally significant (t-ratio = 1.861, df = 166, *p* = 0.06). In the low complexity landscape (LC), no differences were observed between pulses (t-ratio = -1.539, df = 166, *p* = 0.13). Mortality did not exceed 6% in any of the Control groups for either Mospilan or Sherpa exposure.

### Acetylcholinesterase activity after exposure to Sherpa

**Fig. 3:**
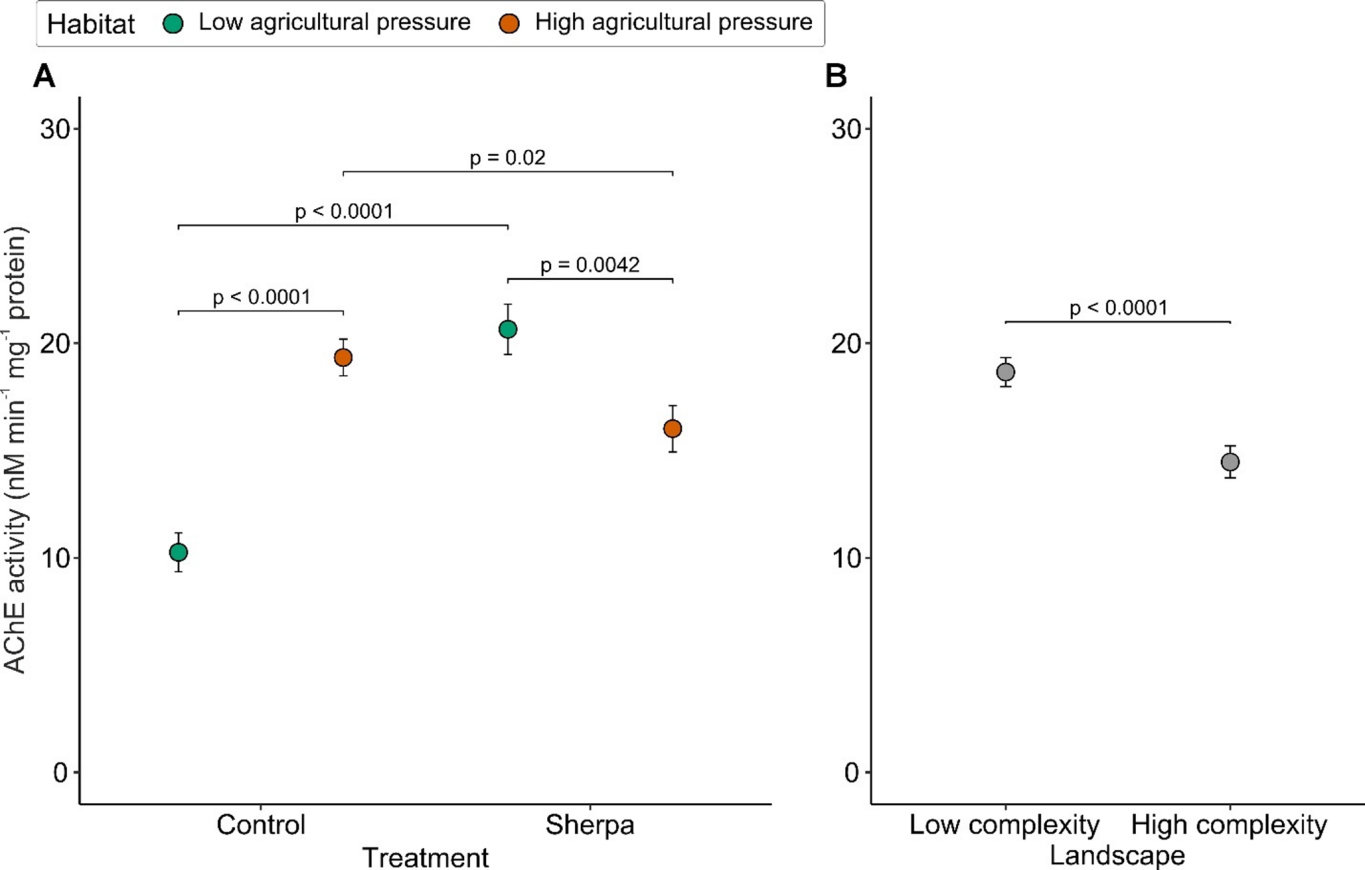
Acetylcholinesterase (AChE) activity (nano moles min^− 1^ mg^− 1^ protein) in *Poecilus cupreus* beetles (F2 generation) exposed to two pulses of 48 h LD_50_ concentration of Sherpa (1.2 RCF; 0.12 µg a.i. × µL^− 1^; a.i. cypermethrin) or control (acetone) treatments. (**A**) Activities for beetles originated from habitats of low (LP) and high (HP) agricultural pressure from control (*n* = 46 and *n* = 49) and insecticide treatments (*n* = 26 and *n* = 31), respectively. (**B**) Activities for beetles from low complexity (LC) (*n* = 82) and high complexity (HC) (*n* = 70) landscapes. P-values are derived from post-hoc analyses calculating estimated marginal means. Points represent estimated marginal means values and bars represent standard errors. RCF – Recommended Concentration for Field use

Top-ranked GLMs (generalized linear models) for AChE activity in beetles exposed to Sherpa are presented in Table S4. Acetylcholinesterase activity after exposure to Sherpa was influenced by: (1) habitat type (χ2 = 14.688, df = 1, *p* = 0.0001); (2) landscape type (χ2 = 17.448, df = 1, *p* < 0.0001); (3) treatment (χ2 = 9.375, df = 1, *p* = 0.002); and (4) interaction of habitat type and treatment (χ2 = 46.163, df = 1, *p* < 0.0001) (Table 2). Post hoc analysis of the interaction between habitat type and treatment showed that basal AChE activity of control beetles differed significantly between habitat types, with beetles from habitats of high (HP) versus low (LP) agricultural pressure having on average 1.7 times higher activity (t-ratio = -11.560, df = 147, *p* < 0.0001; Fig. 3A). Exposure to Sherpa caused a strong, 2-fold increase in AChE activity in beetles from habitats of low agricultural pressure (LP) (t-ratio = 7.479, df = 147, *p* < 0.0001, Fig. 3A). In contrast, beetles from habitats of high agricultural pressure (HP) had moderately reduced AChE activity after exposure to Sherpa (t-ratio = -6.032, df = 147, *p* = 0.02, Fig. 3A). As a result, exposed beetles from habitats of high agricultural pressure (HP) had on average 25% lower AChE activity compared to beetles from habitats of low agricultural pressure (LP) (t-ratio = 1.489, df = 147, *p* = 0.0042, Fig. 3A). The AChE activity in beetles from the low complexity landscape (LC) was significantly higher than that in beetles from the high complexity landscape (HC), with a difference of ca. 15% (Fig. 3B).

**Table 2:** Likelihood ratio chi-square statistics (χ²) for the final generalized linear model (GLM) examining acetylcholinesterase (AChE) activity in *Poecilus cupreus* beetles from different habitat and landscape types after pulse exposure to the insecticide Sherpa. The model includes habitat type, landscape type, treatment (Sherpa exposure), and the interaction between habitat type and treatment. Significance levels: *p < 0.5, **p < 0.01, ***p < 0.001

AChE	χ2	df	*p*-value	
Habitat type	14.688	1	0.0001	***
Landscape type	17.448	1	< 0.0001	***
Treatment	9.375	1	0.002	**
Habitat type × Treatment	46.163	1	< 0.0001	***

Note: df – degrees of freedom

## Discussion

In the exploration of the intricate relationship between landscape structure and the effects of pesticide applications, our study reveals a dynamic interplay across both temporal and spatial dimensions. The multifaceted nature of this interaction is underpinned by the complex dynamics governing animal behaviour, ecological relationships, and exposure patterns (Topping et al. 2015). Our results demonstrate that historical pesticide exposure, shaped by landscape complexity can contribute to divergent sensitivity among non-target arthropod populations. These patterns may reflect early stages of resistance evolution, potentially involving mechanisms such as enhanced detoxification or altered target-site sensitivity (Clements et al. 2020; Fournier and Mutero 1994; Gunning and Moores 2001). While such adaptations are well studied in pest species, their occurence in beneficial non-target organisms remains poorly understood (Brans et al. 2021; Sowa et al. 2022). These lineage-specific responses must also be viewed in a broader spatial context. The principle of ‘action at a distance’ becomes particularly relevant, as in-crop mortality can give rise to off-crop effects that ripple across the landscape (Topping et al. 2020). Both empirical studies and simulation models highlight the central role of landscape-scale patterns of land use and complexity in shaping how agricultural practices, especially pesticide applications, affect non-target arthropod abundance and community composition (Muneret et al. 2023; Topping et al. 2015).

We have shown that agricultural landscape complexity and intensification of agricultural management may influence insecticide susceptibility in future generations of the non-target arthropod *P. cupreus*. This includes exposure to relatively high levels of Mospilan (9.2 RCF; 0.74 µg a.i. × µL^− 1^) as well as close to field levels of Sherpa (1.2 RCF; 0.12 µg a.i. × µL^− 1^), representing 48 h LD_50_ or 48 h near-LD_50_ values. We found partial support for the pulse application hypothesis (i): survival declined after the second pulse of Mospilan regardless of habitat or landscape type, likely due to high insecticide concentration (9.2 RCF), which exceeds typical field levels. In turn, although marginally significant, beetles exposed to field-realistic concentration of Sherpa (1.2 RCF) showed a 23% increase in survival after the second pulse in the high complexity landscape, suggesting rapid recovery after the first pulse. Thus, the slow recovery after the second pulse of Mospilan was most likely due to a relatively high concentration of insecticide, which should not be present in agricultural fields under normal circumstances. The survival and physiological responses of *P. cupreus* to field-realistic Sherpa exposure highlight the potential protective role of habitat heterogeneity in mitigating the negative insecticide effects, even after two generations of laboratory rearing. Sustained elevated AChE activity found in beetles from the low complexity landscape, did not translate into improved survival after pulsed exposure to Sherpa, suggesting potential physiological costs associated with increased enzyme activity. Conversely, lower AChE activity and higher survival of beetles originating from the high complexity landscape suggest that such an environment provides refuges and resources that alleviate stress and support recovery from pulse exposure. Importantly, repeated low-dose pesticide applications may pose cumulative risks to invertebrate populations (Sánchez-Bayo and Tennekes 2020; Wiberg-Larsen et al. 2021), yet studies on beneficial terrestrial arthropods under pulsed insecticide exposure remain scarce. Evidence from aquatic ecosystems (Betz-Koch et al. 2024; Wiberg-Larsen et al. 2021), as well as our own findings, highlight the urgent need for research in terrestrial agroecosystems, particularly considering the role of landscape complexity.

Our results suggest that local habitat and landscape context jointly influence insecticide susceptibility, with effects persisting in beetle lineages reared for two generations under laboratory conditions. Our hypothesis (ii) that beetles from habitats of high agricultural pressure would have a higher probability of survival was supported for both insecticides tested, but only for beetles from low complexity landscapes. Similarly, our hypothesis (iii) that reduced susceptibility to insecticides would be more evident in beetles from low complexity landscape was only supported for beetles exposed to Sherpa originated from habitats of high agricultural pressure. Similar results were obtained by Sowa et al. (2022), who observed reduced susceptibility to Proteus 110 OD in the second generation of *P. cupreus* beetles for which the parental generation was collected from high pressure habitats within low complexity landscape. Proteus 110 OD insecticide contains thiacloprid and deltamethrin, belonging to the same neonicotinoid and pyrethroid groups as the active ingredients in Mospilan and Sherpa. Our results, combined with those of Sowa et al. (2022), suggest that reduced susceptibility in beetles may require long-term pesticide pressure at both local and landscape scales, especially in landscapes of low complexity under high agricultural pressure. However, it is important to note that the observed effects may be confounded by the chemical properties of tested insecticides or applied doses.

Low complexity landscapes, with limited habitat availability and connectivity, restrict beetle movement and reduce genetic exchange between populations, leading to lower genetic diversity (Gauffre et al. 2015). As a result, small, fragmented beetle populations from habitats with permanently low agricultural pressure embedded in such landscapes may therefore be more vulnerable to stressors such as insecticides (Bijlsma and Loeschcke 2012). This may explain the lowest survival rates after exposure to Mospilan, and one of the lowest to Sherpa, among the beetles originated from habitats of low agricultural pressure within low complexity landscape. Thus, in low complexity landscapes, isolated populations are more dependent on local conditions than in more complex landscapes. Such populations may exhibit a ‘tipping point’ (Dakos et al. 2019), i.e. as local agricultural pressure increases, the beetle may at some point start to develop reduced susceptibility to insecticides if the pressure remains constant over a long period of time. Thus, the persistence of reduced susceptibility to neonicotinoids and pyrethroids over generations observed in our study and by Sowa et al. (2022) in high pressure habitats within low complexity landscape may indicate the development of resistance or selection for more resistant individuals due to continuous insecticide exposure under intensive agricultural management (Georghiou 1972). While the mechanism of reduced susceptibility is beyond the scope of the current study, it likely involves a combination of genetic, metabolic, physiological, and behavioural adaptations, as suggested by Bras et al. (2022).

Highly complex landscapes are thought to promote biodiversity and the abundance of non-target species, potentially mitigating the negative effects of intensive agricultural practices (Gámez-Virués et al. 2015; Martin et al. 2019; Ziółkowska et al. 2021, 2022). Therefore, beetles from more complex landscapes may benefit from better access to recovery sites, which may explain the lack of differences in survival of beetles from low and high pressure habitats that we found in the high complexity landscape after exposure to Mospilan, and even moderately higher survival of beetles from low than high pressure habitats in the high complexity landscape after exposure to Sherpa. Since the beetles were bred in the laboratory for two generations, these patterns likely reflect inherited differences shaped by environmental history, rather than immediate habitat effects. This raises the possibility that the mitigating effects of landscape complexity may, in certain cases, be strong enough to reduce the negative effects of agricultural pressure on beetle survival (Roubos et al. 2014). We acknowledge that in our study landscape complexity was represented by only one study area per category, and thus this factor was not statistically replicated at the landscape level. As a result, any interpretation of landscape effects should be made with caution, as we cannot fully disentangle them from other potential regional influences. Nevertheless, the landscape contrast was deliberately chosen to reflect ecologically meaningful differences in farmland structure, serving as a proxy for environmental history shaping lineage-specific responses.

In addition to investigating direct (acute) effects after exposure to insecticides, we also investigated biochemical responses by measuring AChE activity (a known biomarker of pesticide exposure) in beetles exposed to Sherpa and from control treatments. The AChE activity in control beetles, which represent the ‘baseline’ activity for both habitat types, was higher in beetles originated from habitats of high agricultural pressure, supporting our hypothesis (iv). A similar hypothesis regarding increased AChE activity in *P. cupreus* beetles collected from habitats of high agricultural pressure (where pressure was measured by oilseed rape coverage in local habitat) was previously rejected by Sowa et al. (2023). The fact that the ‘baseline’ AChE activity is higher in beetles from habitats of high agricultural pressure may indicate that these beetles have developed some adaptive physiological response over several in-field generations to cope with frequent exposure to insecticides (Mohamadi et al. 2010). As beetles in our experiment were derived from a second-generation laboratory population reared under controlled, pesticide-free conditions, our setup minimizes the likelihood of direct or maternal effects (Lagisz and Laskowski 2008). Furthermore, observed mortality where the most resistant individuals survived, may explain why Sowa et al. (2023) observed less pronounced differences. Overall, this is consistent with the concept that higher baseline AChE activity may be a protective adaptation that allows beetles to rapidly detoxify or degrade insecticides upon exposure.

Interestingly, the observed pattern of AChE activity in control beetles changed after pulse exposure to Sherpa with higher AChE activity observed in beetles from habitats of low agricultural pressure. This may indicate that beetles with limited historical pesticide exposure are more sensitive to insecticides and may exhibit a temporary increase in AChE activity as an acute stress response. This aligns with the findings of Badiou and Belzunces (2008), who reported increased AChE activity in bees exposed to deltamethrin and its mixture with pirimicarb. However, the suitability of AChE activity as a biomarker of pyrethroid exposure remains uncertain, as pyrethroids primarily target sodium channels rather than directly inhibiting AChE activity. Thus, the observed increase in AChE activity may reflect a non-specific stress response rather than AChE inhibition per se. AChE activity in beetles after exposure to Sherpa was not influenced by the interaction between habitat and landscape type, suggesting these factors act independently. For example, agricultural pressure may directly select for higher AChE in beetles (Fournier and Mutero 1994; Gunning and Moores 2001), while landscape complexity may influence beetle fitness or survival through other mechanisms than detoxification enzyme activity (Tiede et al. 2022). Interpreting enzyme activity changes requires caution, as AChE activity/insensitivity is often linked with other resistance mechanisms (Ishaaya 2012) and influenced by factors like genetic variability and environmental factors beyond agricultural pressure. However, maintaining elevated biochemical activity can be energetically costly, shifting from vital processes such as reproduction, which may compromise provision of ecosystem services (Calow 1991).

Our findings suggest that Ecological Risk Assessment (ERA) frameworks could be strengthened by incorporating environmental history, physiological biomarkers, and lineage-based responses. Traditional ERA approaches, which rely heavily on acute, short-term toxicity tests, may overlook important factors such as historical pesticide exposure, landscape context, and evolutionary adaptation (Levine et al. 2019). Consistent with recent concerns raised by Boyd et al. (2025), our results indicate that standard laboratory test species that may not adequately reflect environmental complexity or evolutionary history. This highlights the importance of ecotoxicological designs that consider wild origins and multigenerational exposure contexts. In our study, differences in susceptibility and AChE activity observed after two laboratory generations suggest that environmental origin can influence pesticide sensitivity in ways that standard tests may not reveal. Recent methodological advances advocate for landscape-based ERA approaches that incorporate spatial heterogeneity and source–sink dynamics in shaping population responses to pesticides (Tarazona et al. 2024). Our data support these perspectives, indicating that higher-tier assessments integrating multigenerational, ecologically contextualized assays — with physiological endpoints (e.g., enzyme activity) and landscape-informed environmental histories — may provide more realistic predictions of long-term risks to non-target organisms in agricultural systems.

## Conclusions

This study highlights the complex interplay between landscape structure, local habitat pressures, and non-target arthropod susceptibility to pesticides, using *P. cupreus* beetle as example. Our results indicate that landscape complexity plays a crucial role in shaping beetle survival and adaptation to insecticides such as Mospilan and Sherpa. We showed that high complexity landscape, characterized by increased edge density and spatial diversity, supports better beetle recovery from insecticide exposure. Furthermore, beetles from low complexity landscape under high local agricultural pressure showed reduced susceptibility compared to those under low local agricultural pressure, likely due to multi-generational pesticide exposure and potential resistance evolution. However, these adaptations may come at a metabolic cost, compromising reproductive success and ecosystem function.

Our findings suggest that beetles from habitats of high agricultural pressure may have developed a physiological adaptation, reflected by higher baseline AChE activity, which may allow them to detoxify insecticides more efficiently. In contrast, beetles from habitats of low agricultural pressure exhibited elevated AChE activity after pulse exposure to Sherpa, possibly as a temporary response to acute stress. This highlights that while habitat and landscape complexity influence AChE activity, they do so through separate mechanisms, underscoring the complexity of using AChE as the sole biomarker for assessing insecticide sensitivity in different environments.

Finally, our findings suggest that ERA protocols could benefit from incorporating multigenerational, lineage-based designs together with biomarker data. Traditional short-term assays may not fully capture the evolutionary and physiological processes that shape non-target species’ susceptibility. Integrating environmental history, physiological markers, and landscape context may enhance both the predictive power and ecological realism of pesticide risk assessments and the ecotoxicological testing.

## Supplementary Information

Below is the link to the electronic supplementary material.

Supplementary Material 1

Supplementary Material 2

## Data Availability

Raw dataset is available in Supplementary Information.
