# Supplementary material for: Landscape complexity and agricultural pressure at the habitat-level scale determine impact of insecticide use on future generation of non-target arthropods (ground beetle Poecilus cupreus)
Source: Ecotoxicology. 2026 Jan 3;35(2):22. doi: 10.1007/s10646-025-02995-5 (PMC12764627; doi:10.1007/s10646-025-02995-5)
Supplement: Supplementary file 1 — Supplementary Material 1 [file 10646_2025_2995_MOESM1_ESM.pdf]

ECOTOXICOLOGY JOURNAL - SUPPLEMENTARY MATERIAL FOR:

Landscape complexity and agricultural pressure at the habitat-level scale determine impact of insecticide use on future generation of non-target arthropods (ground beetle *Poecilus cupreus*)

Grzegorz Sowa [a] *, Elżbieta Ziółkowska [a], Zuzanna M. Filipiak [a] *

[a] Institute of Environmental Sciences, Jagiellonian University, Gronostajowa 7, 30-387 Krakow, Poland

ORCID numbers:

Grzegorz Sowa - 0000-0001-7855-0545

Elżbieta Ziółkowska - 0000-0002-7213-2200

Zuzanna M. Filipiak - 0000-0001-5843-5195

*__Corresponding authors__:

Zuzanna M. Filipiak

Email: zuzanna.filipiak@uj.edu.pl

Phone: (+48)126646878

Fax: (+48)126646912

Grzegorz Sowa

Email: grzegorz.sowa@uj.edu.pl

Phone: (+48)126645133

Fax: (+48)126646912

Materials and methods

Research area

The area from which the parental generation (P) of beetles were collected is located in western Poland and represents a typical farmland with approximately 65-70% arable land coverage, strongly dominated by conventional farming. Within the area, two different landscapes were selected based on the farmland's structure (Table S1). Both landscapes were similar in terms of land cover and pedo-climatic conditions but differed considerably in terms of number of fields and their size classes. Detailed land cover / land use raster maps (resolution of 1 m^2) for the landscapes were generated using the methodology presented in Ziółkowska et al. (2021) and Sowa et al. (2022). With information from the Land Parcel Identification System (LPIS) for Poland for the year 2018, integrated with the Topographic Objects Database (BDOT10k), spatial patterns of cultivated crops across the analyzed landscapes were generated. Based on these patterns, each study area was characterized by measures of landscape and farmland composition (assessed for the year 2018) important from the point of view of analyzed species. *Poecilus cupreus* prefers open habitats (arable fields, permanent pastures and meadows) over linear grassy elements (fields margins and road verges) and can be found only sporadically in woody vegetation (forest and bushes) (Marrec 2015, Barone and Frank 2003, Anjum-Zubair et al. 2010). It is also known to inhabit areas close to infrastructure such as parklands and even domestic gardens (Braschler et al. 2020) (Table S2).

Table S1. Two study areas, denoted as either low complexity landscape or high complexity landscape, with share of arable land in given field size classes [%] and total number of arable fields. Taken from Sowa et al. (2022).

Feature	Field size classes (ha)	Low complexity landscape	High complexity landscape
Share of arable land [%] in given field size classes	< 3 ha	23.5	41.7
	3–10	25.7	40.1
	10–30	21.7	11.5
	30–50	16.8	4.8
	≥ 50	12.3	1.9
Total number of arable fields		4072	6494

Table S2. Location and characteristics of the study sites within analyzed landscapes, including measures of landscape and farmland composition (assessed for the year 2018) important from the point of view of analyzed species. The beetles originated from two landscapes that differ in complexity, i.e., one with more homogenous farmland, dominated by medium and large fields managed mostly by large agricultural holdings (denoted as 'low complexity' landscape; LC) and the other, more heterogenous one, dominated by small fields belonging mostly to family farms (denoted as 'high complexity' landscape; HC), as well as from two habitat types that differed in agricultural pressure on beetles (i.e., 'high agricultural pressure' (HP) and 'low agricultural pressure' (LP)).

Site ID	Landscape type	Habitat type	Longitude (decimal degrees)	Latitude (decimal degrees)	Landscape composition (% of coverage)						Farmland composition (% of coverage)		
					Arable land	Permanent pastures	Meadows	Linear grassy elements[1]	Forest and bushes	Vegetation close to infrastructure[2]	Winter oilseed rape	Winter wheat	Winter triticale
**1**	HC	HP	17.2919	51.7944	98.4	0.1	0.0	0.8	0.4	0.0	52.4	5.3	0.0
**2**	HC	HP	17.3729	51.8070	92.0	0.3	1.3	1.5	0.8	0.7	33.9	0.0	5.8
**3**	HC	HP	17.2874	51.7292	64.0	22.7	1.2	0.7	0.9	3.9	17.7	0.0	3.4
**4**	HC	HP	17.2597	51.7373	82.4	0.4	2.0	1.5	3.8	5.1	17.2	2.4	3.4
**5**	HC	HP	17.3382	51.8126	90.9	0.0	0.0	1.0	0.9	3.6	14.1	0.6	0.7
**6**	HC	HP	17.3253	51.8199	94.9	0.0	0.0	1.9	0.5	0.3	10.4	0.6	2.9
**7**	HC	LP	17.3959	51.8162	73.3	7.4	14.4	0.6	0.9	0.0	0.0	2.9	20.5
**8**	HC	LP	17.2615	51.7249	57.7	18.8	3.6	1.6	4.7	5.8	0.0	0.0	0.7
**9**	HC	LP	17.3525	51.7569	53.7	28.0	3.4	1.1	1.1	7.0	0.0	6.9	3.9
**1**	LC	HP	16.7829	52.0563	97.4	0.0	0.0	1.4	0.4	0.0	100.0	0.0	0.0
**2**	LC	HP	16.8044	52.0287	92.2	0.0	0.1	0.3	5.0	0.0	84.2	0.0	0.0
**3**	LC	HP	16.9091	52.1349	90.1	4.5	0.2	0.9	1.8	0.4	97.1	0.0	0.0

**4**	LC	HP	16.8475	52.1148	96.1	0.1	0.0	1.6	0.6	0.0	32.6	2.6	15.8
**5**	LC	HP	16.8688	52.1462	95.1	0.0	0.0	1.1	2.0	0.0	41.8	0.0	0.1
**6**	LC	HP	16.8478	52.1396	67.9	8.4	17.0	0.6	0.8	0.4	38.6	0.0	0.0
**7**	LC	LP	16.8568	52.1341	62.0	0.0	24.3	0.6	2.7	1.5	0.0	0.0	0.0
**8**	LC	LP	16.8140	52.0627	45.7	4.6	12.4	1.6	5.8	16.8	0.0	0.0	69.9
**9**	LC	LP	16.8259	52.0864	65.6	5.8	4.5	2.0	4.6	1.8	0.0	0.0	0.0

[1] Linear grassy elements = grassy field margins and road verges

[2] Vegetation close to infrastructure = parkland, garden, and amenity grass

Selection of pesticides and the exposure concentrations

To determine which pesticides were the most commonly used in the research area where the beetles were collected, we conducted a survey among oilseed rape farmers. We learned that the most commonly used insecticides products contained acetamiprid, chlorpyrifos, a mixture of thiacloprid and deltamethrin, or several different pyrethroids (e.g., cypermethrin or zeta-cypermethrin) as an active ingredient. Based on the survey, we chose Mospilan® SP 20 with acetamiprid (20%) and Sherpa® 100 EC with cypermethrin (100 g/L). The acetamiprid belongs to the neonicotinoid insecticide group and acts as an agonist of the post-synaptic nicotinic acetylcholine receptors (nAChRs) of insects (Tomizawa and Casida 2005). The neonicotinoids interact with the nAChR in a structure-activity relationship resulting in paralysis (Tomizawa and Casida 2005). Cypermethrin, in turn, is a pyrethroid that acts on the voltage-dependent sodium channels in the excitable membranes of the nervous system causing membrane depolarization followed by excitation (abnormal hyperexcitability) due to the protraction of the sodium current (Badiou and Belzunces 2008). The hyperexcitability overwhelms the cell's ability to maintain the activity of the sodium pump and, as a result, insects become paralyzed (knocked down) (Davies et al. 2007).

The preliminary experiment was carried out on the ground beetle *P. cupreus*. The beetles belonged to the group of the laboratory cultured individuals (generation F2) originated from habitats of low agriculture pressure (LP) in the high complexity landscape (HC). Before exposure to insecticides, the beetles were kept for 24 h in the plastic cups with perforated lids and moisturize filter paper Petri dishes to acclimatise to laboratory conditions: 20 °C and 75% relative humidity (RH) under a 16:8 (light:dark; L:D) h cycle. The beetles (n = 130), ten individuals per treatment, were randomly assigned to twelve pesticide treatments and a control. Six exposure concentrations were chosen for each of the pesticides studied, corresponding to 0.65; 1.3; 2.5; 5; 10; 20 recommended concentrations for field use. For insecticides dissolved

in acetone, beetles were exposed individually to 1 µl of pesticide solution, whereas individuals from control groups were treated with acetone alone. Doses were applied on the scutellum using a repeating topical dispenser attached to Hamilton syringe (Hamilton company; USA). Perti dishes were kept at 20 °C and 75% RH under a 16:8 (L:D) h cycle. Beetles were not fed during the experiment. Mortality and immobility (recognized as the knock down effect) were recorded after 0.5; 1; 2; 4; 6; 8; 10; 12. 24 and 48 h. Insects were classified as dead or knocked down (paralyzed) after gentle prodding with a pair of tweezers followed by observation of their movements. When beetles were observed to be knocked down (unable to walk/relocate), they were treated as dead. From the mortality results, the LD_{50} (48 h) for each insecticide was estimated with 95% confidence intervals using probit analysis. Statistical analyses were performed using Statgraphics Centurion 18 (Statgraphics Technologies. Inc., USA).

Results

Preliminary experiment

Table S3. Lethal doses (LD) causing 50% mortality, corresponding to the range of tested recommended field doses calculated using probit analysis for results of *Poecilus cupreus* beetle mortality after 48-h exposure to commercial formulation of pesticides acetamiprid (Mospilan® SP – Nippon Soda, Japan) and cypermethrin (Sherpa® 100 EC – SBM Developpement SAS, France). CI – 95% confidence intervals for LD_{50}

**Pesticide**	**LD_{50}**	**CI (95%)**	**Percentage of deviance explained by model**
Mospilan® SP 20	9.2	4.3 – 23.2	25.7
Sherpa® 100 EC	1.2	0.9 – 1.6	99.9

Table S4. Top ranking generalized linear models (GLMs; estimates and model weights) for beetle AChE activity exposed to Sherpa. Models are listed in descending order according to their ΔAICc. Models with ΔAICc < 4 are presented. Parameter estimates, degrees of freedom (df), log-likelihood (LL), Akaike's Information Criterion with small-sample size adjustment (AICc), ΔAICc, Akaike weights (w_i), and R^2 are reported.

Intercept	hbt	lnd	trt	hbt:lnd	hbt:trt	lnd:trt	df	LL	AICc	ΔAICc	w_i	R^2
21.42	+	+	+		+		6	-484.87	982.3	0.00	0.519	0.347
21.83	+	+	+	+	+		7	-484.57	983.9	1.61	0.232	0.350
21.43	+	+	+		+	+	7	-484.87	984.5	2.20	0.173	0.347
21.83	+	+	+	+	+	+	8	-484.57	986.2	3.84	0.076	0.350

The plus sign (+) indicates the inclusion of the variable in the considered model, colon (:) indicates interaction between variables, hbt – habitat type, lnd – landscape type, trt – treatment. We selected the top-ranked model built for beetles exposed to Sherpa with an AICc weight of 52% and with evidence ratio of 2.2 relative to the next ranked model.

References

Anjum-Zubair M, Schmidt-Entling M, Querner P, Frank T (2010) Influence of within-field position and adjoining habitat on carabid beetle assemblages in winter wheat. Agricultural and Forest Entomology 12(3):301. https://doi.org/10.1111/j.1461-9563.2010.00479.x

Badiou A, Belzunces LP (2008) Is acetylcholinesterase a pertinent biomarker to detect exposure of pyrethroids? A study case with deltamethrin. Chem Biol Interact 175:406–409. https://doi.org/10.1016/j.cbi.2008.05.040

Barone M, Frank T (2003) Habitat age increases reproduction and nutritional condition in a generalist arthropod predator. Oecologia 135:78-83. https://doi.org/10.1007/s00442-002-1175-2

Braschler B, Gilgado JD, Zwahlen V, Rusterholz HP, Buchholz S, Baur B (2020) Ground-dwelling invertebrate diversity in domestic gardens along a rural-urban gradient: Landscape characteristics are more important than garden characteristics. PloS one 15(10):e0240061. https://doi.org/10.1371/journal.pone.0240061

Davies TGE, Field LM, Usherwood PNR, Williamson MS (2007) DDT, pyrethrins, pyrethroids and insect sodium channels. IUBMB Life 59:151–162. https://doi.org/10.1080/15216540701352042

Marrec R, Badenhausser I, Bretagnolle V, Börger L, Roncoroni M, Guillon N, Gauffre B (2015) Crop succession and habitat preferences drive the distribution and abundance of carabid beetles in an agricultural landscape. Agriculture, Ecosystems & Environment 199:282-289. https://doi.org/10.1016/j.agee.2014.10.005

Sowa G, Bednarska AJ, Ziółkowska E, Laskowski R (2022) Homogeneity of agriculture landscape promotes insecticide resistance in the ground beetle Poecilus cupreus. PLoS One 17:e0266453. https://doi.org/10.1371/journal.pone.0266453

Tomizawa M, Casida JE (2005) Neonicotinoid insecticide toxicology: Mechanisms of selective action. Annu Rev Pharmacol Toxicol 45:247–268. https://doi.org/10.1146/annurev.pharmtox.45.120403.095930

Ziółkowska E, Topping CJ, Bednarska AJ, Laskowski R (2021) Supporting non-target arthropods in agroecosystems: Modelling effects of insecticides and landscape structure on carabids in agricultural landscapes. Sci Total Environ 774:145746. https://doi.org/10.1016/j.scitotenv.2021.145746
